# Modifiable Risk Factors in Hispanic Adults With Gastric Cancer in the United States

**DOI:** 10.7759/cureus.61920

**Published:** 2024-06-07

**Authors:** Alejandro J Nieto Dominguez, Sarah E Eichinger, Daniel Guifarro, Chun-Wei Pan, Bashar Attar

**Affiliations:** 1 Internal Medicine, John H. Stroger, Jr. Hospital of Cook County, Chicago, USA; 2 Gastroenterology, John H. Stroger, Jr. Hospital of Cook County, Chicago, USA

**Keywords:** oncology, hispanic immigrants, hispanic population, hispanic, helicobacter pylori, racial disparities, gastric cancer, gastroenterology

## Abstract

Background: Hispanics make up 19% of the U.S. population and are experiencing rising rates of cancer, primarily due to an increase in infection-related cancers (gastric, hepatic, cervical) and advanced cancers secondary to delayed screening (colorectal, cervical, breast). There is an increased incidence of gastric cancer (associated with infection, obesity, alcohol, and tobacco use) in Hispanics, especially at a young age, highlighting the need to consider ethnicity as a risk factor.

Methods: This study utilized the 2016-2019 National Inpatient Sample database to examine all patients admitted with gastric cancer. Individuals were stratified by race, age, and comorbidities, including modifiable risk factors that are associated with gastric cancer.

Results: There were 5,785 (7.44%) patients aged 18-44, 28,370 (36.49%) aged 45-64, and 43,590 (56.07%) over 65 years of age. Notably, 34.3% of the youngest group were Hispanic, contrasted with 19.7% and 12.9% in the older groups, respectively. Younger Hispanic patients showed a higher prevalence of *H. pylori *infection (8.6%) compared with older Hispanics (3.6% in the middle age group and 2.1% in the oldest, p<0.01). There was a high prevalence of obesity, tobacco use, and gastric ulcers in this cohort. Other risk factors such as alcohol use and gastric polyps were present at a lesser prevalence.

Conclusions: This study reveals that Hispanic patients tend to have a younger age of onset of gastric cancer, coupled with an increased incidence of H. pylori infection at a younger age. This finding underscores the potential benefit of H. pylori screening among asymptomatic young Hispanics with the aim of reducing gastric cancer morbidity and mortality in this population.

## Introduction

Gastric cancer is diagnosed in approximately 990,000 people worldwide each year. In the United States, the incidence rate of gastric cancer is significantly higher in the Hispanic population compared to non-Hispanics [1]. While rates of gastric cancer have been decreasing in the United States, it remains a leading cause of cancer-related deaths worldwide, particularly in East Asian countries, Latin America, and among Hispanic individuals in the United States (who make up 19% of the US population) [2].

The incidence in patients less than 50 years old has been increasing for unknown reasons, particularly among Hispanic individuals. Holowatyj et al. found that in the United States, two out of every five young patients diagnosed with noncardia gastric cancer from 2007-2015 were Hispanic. They also demonstrated that Hispanic patients have a greater risk of having more advanced disease at initial presentation and suffering more complications throughout their treatment course [3].

Risk factors associated with gastric cancer include *Helicobacter pylori *infection, obesity, alcohol, tobacco use, diet, limited physical activity, family history, age, genetics, and history of gastric polyps [4]. *H. pylori* infection, in particular, colonizes the gastric mucosa of hosts, and undetected chronic inflammation may drive transformation to pre-neoplastic and neoplastic lesions [5]. The mechanism of development of neoplastic lesions arises from virulence factors from the bacteria that promote the creation of reactive oxygen species and nitroso compounds, resulting in epithelial damage [6].

Factors such as diverse dietary traditions, unique cooking methods, and access to different foods may result in disparities in rates of gastric cancer within different populations. Additionally, there may be genetic variants between *H. pylori *strains in distinct populations that result in different outcomes [7]. Finally, barriers to care, particularly those faced by minority populations, may lead to an increased risk for gastric cancer and complications. For example, immigrants from Latin America to the United States are faced with underinsurance, language barriers, and difficulties navigating a foreign medical system, potentially contributing to advanced disease on presentation and worse outcomes [8-10]. Given the recent trend of increasing gastric cancer in younger patients, particularly in young Hispanic patients, risk factors and strategies for modification of these risk factors warrant additional investigation.

## Materials and methods

Study design and data source

This retrospective cohort study utilized data from the National Inpatient Sample (NIS) database, spanning the years 2016-2019. The NIS, part of the Healthcare Cost and Utilization Project (HCUP), is the largest publicly available all-payer inpatient healthcare database in the United States. It contains data on hospital inpatient stays, allowing for the analysis of national trends in healthcare utilization, access, charges, quality, and outcomes. The NIS database provides a stratified sample of hospitals across the United States, including public hospitals, academic medical centers, and private hospitals, ensuring a representative sample of the U.S. inpatient population [11].

Inclusion and exclusion criteria

The inclusion criteria for this study were as follows: (1) patients aged 18 and older; hospitalized patients with a primary diagnosis of gastric cancer, identified using ICD-10 codes (C16, C16.0, C16.1, C16.2, C16.3, C16.4, C16.5, C16.6, C16.8, C16.9) (Table 1); patients with a secondary diagnosis of any of the following: *H. pylori* infection, history or current tobacco use, alcohol use, gastric ulcer, gastric polyp, and obesity (see Table 2 for their respective ICD-10 codes); and patients with documented race/ethnicity (White, Black, Hispanic, Asian/Pacific Islander). The exclusion criteria included patients with secondary or metastatic gastric cancer as the primary diagnosis and patients under 18 years of age.

**Table 1:** Gastric cancer-related ICD-10 codes

ICD-10 codes	Description
C16	Malignant neoplasm of the stomach
C16.0	Malignant neoplasm of the cardia
C16.1	Malignant neoplasm of the fundus of the stomach
C16.2	Malignant neoplasm of the body of the stomach
C16.3	Malignant neoplasm of the pyloric antrum
C16.4	Malignant neoplasm of the pylorus
C16.5	Malignant neoplasm of the lesser curvature of the stomach, unspecified
C16.6	Malignant neoplasm of the greater curvature of the stomach, unspecified
C16.8	Malignant neoplasm of overlapping sites of the stomach
C16.9	Malignant neoplasm of the stomach, unspecified

Study variables and outcomes

The primary outcome was the prevalence of modifiable risk factors associated with gastric cancer among different racial/ethnic groups. The risk factors included obesity, gastric polyps, gastric ulcers, *H. pylori *infection, alcohol use, and history or current tobacco use. The ICD-10 codes used for identifying these risk factors are given in Table 2.

**Table 2:** Modifiable risk factors for gastric cancer-related ICD-10 codes

Risk factor	ICD-10 codes
History or current tobacco use	Z87.891, F17, Z72.0
Obesity	E66.01, E66.09, E66.1, E66.2, E66.8, E66.9, O99.2, Z68.3, Z68.4, Z68.54
Gastric polyp	K31.7
Gastric ulcer	K25.9, K25, K25.0, K25.1, K25.2, K25.3, K25.4, K25.5, K25.6, K25.7
*Helicobacter pylori *infection	B96.81
Alcohol use	F10, F10.1, F10.10, F10.11, F10.12, F10.120, F10.121, F10.129, F10.13, F10.130, F10.131, F10.132, F10.139, F10.14, F10.15, F10.150, F10.151, F10.159, F10.18, F10.180, F10.181, F10.182, F10.188, F10.19, F10.2, F10.20, F10.21, F10.22, F10.220, F10.221, F10.229, F10.23, F10.230, F10.231, F10.232, F10.239, F10.24, F10.25, F10.250, F10.251, F10.259, F10.26, F10.27, F10.28, F10.280, F10.281, F10.282, F10.288, F10.29, F10.9, F10.90, F10.91, F10.92, F10.920, F10.921, F10.929, F10.93, F10.930, F10.931, F10.932, F10.939, F10.94, F10.95, F10.950, F10.951, F10.959, F10.96, F10.97, F10.98, F10.980, F10.981, F10.982, F10.988, F10.99

Statistical analysis

Statistical analysis was performed using STATA MP 18.0. Descriptive statistics were used to summarize patient demographics and the prevalence of risk factors. Categorical variables were expressed as frequencies and percentages. Chi-square tests were used to compare the prevalence of risk factors among different age and racial/ethnic groups. A p-value of <0.05 was considered statistically significant.

Ethical considerations

This study utilized deidentified data from the NIS database and, therefore, was exempt from institutional review board (IRB) approval. All analyses were conducted in accordance with the ethical standards set forth by the Healthcare Cost and Utilization Project (HCUP) data use agreement.

## Results

**Figure 1:**
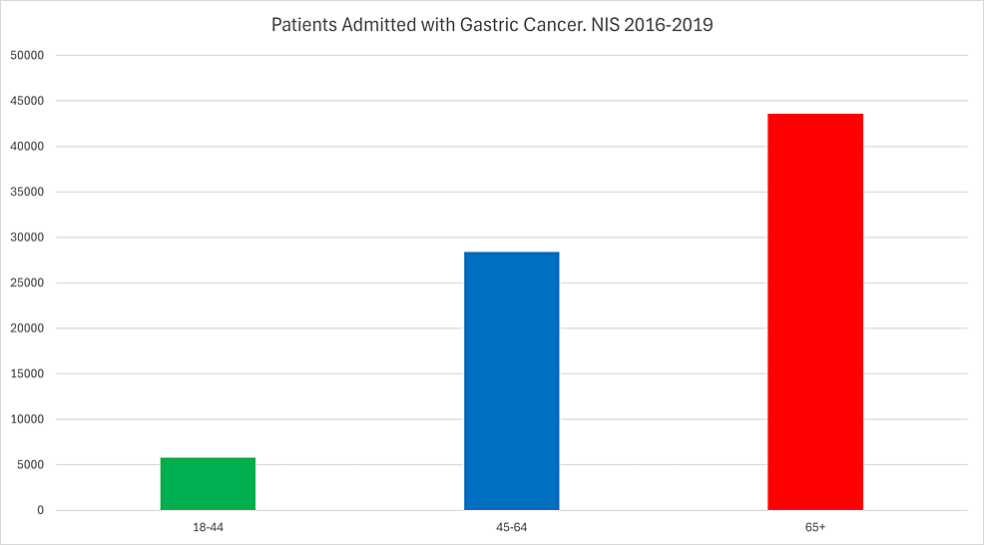
Patients admitted with gastric cancer, NIS 2016-2019 NIS: National Inpatient Sample.

There were 77,745 adult patients with gastric cancer studied. Of these patients, 5785 (7.44%) were between the ages of 18 and 44 years (group 1), 28,370 (36.49%) were between 45 and 64 years (group 2), and 43,590 (56.07%) were over the age of 65 (group 3) (see Figure 1).

Group 1 comprised 1700 (29.4%) White, 1986 (34.3%) Hispanic, 839 (14.5%) Black, and 1260 (21.8%) Asian or Pacific Islander patients. Group 2 consisted of 13,238 (46.7%) White, 5597 (19.7%) Hispanic, 4937 (17.4%) Black, and 4598 (16.2%) Asian or Pacific Islander individuals. Group 3 had the highest percentage of White patients with 24,485 (56.0%) and the lowest percentage of Hispanic individuals with 5623 (12.9%). There were 6588 (15.1%) Black patients and 6934 (15.9%) Asian or Pacific Islander patients. See Table 3 for the demographic characteristics of patients admitted with gastric cancer.

**Table 3:** Gastric cancer rates stratified by age and race

	Group 1 (age 18-44)	Group 2 (age 45-64)	Group 3 (age 65+)
Total number of gastric cancer cases	5785 (7.44%)	28,370 (36.49%)	43,590 (56.07%)
Race			
White	1700 (29.4%)	13,238 (46.7%)	24,485 (56.0%)
Black	839 (14.5%)	4937 (17.4%)	6588 (15.1%)
Hispanic	1986 (34.3%)	5597 (19.7%)	5623 (12.9%)
Asian or Pacific Islander	1260 (21.8%)	4598 (16.2%)	6934 (15.9%)

Analysis of modifiable risk factors for gastric cancer in Hispanic patients demonstrated a predominance of H. pylori among younger Hispanics with gastric cancer: 8.6% of Hispanic patients in Group 1 versus 3.6% and 2.1% in Groups 2 and 3, respectively (p <0.01). The incidence of smoking increased with age, with 19.9% in Group 1, 28.1% in Group 2, and 31.1% in Group 3 (p <0.01). Alcohol use was 3.0% in Group 1, 4.3% in Group 2, and 2.3% in Group 3 (p=0.0348). There was no statistically significant difference in rates of gastric cancer, gastric polyps, or obesity across all groups (see Table 4).

**Table 4:** Modifiable risk factors for gastric cancer present in Hispanic patients

Modifiable risk factors for gastric cancer present in Hispanic patients	Group 1 (18-44)	Group 2 (45-64)	Group 3 (65 or more)	P Value
Helicobacter pylori	171 (8.6%)	202 (3.6%)	118 (2.1%)	<0.01
History or current tobacco use	396 (19.9%)	1576 (28.1%)	1363 (31.1%)	<0.01
Alcohol use	60 (3.0%)	241 (4.3%)	101 (2.3%)	0.034
Gastric ulcer	231 (11.59%)	531 (9.46%)	469 (10.69%)	0.41
Gastric polyp	10 (0.50%)	35 (0.63%)	66 (1.51%)	0.149
Obesity	166 (8.31%)	386 (6.88%)	398 (9.08%)	0.145

In contrast, other racial groups had lower rates of H. pylori infection associated with gastric cancer. Among White patients studied, there was no statistical difference between all age groups, with a low prevalence overall of H. pylori infection (2.35% in Group 1, 1.28% in Group 2, and 1.08% in Group 3, p=0.107). In Black patients studied, the difference in H. pylori infection associated with gastric cancer was non-significant across all age groups, with 3.57% of patients in Group 1, 2.64% in Group 2, and 2.43% in Group 3 (p=0.68). Asian/Pacific Islander patients studied had a prevalence of H. pylori and gastric cancer of 3.97% in Group 1, 2.72% in Group 2, and 1.51% in Group 3 (p=0.01). 

The remainder of the groups had a significant association of gastric cancer with a history of tobacco use. The prevalence of alcohol use was less than 5% in all age groups across all races, except in Group 2 of Black patients, which had a prevalence of 8.01%.

Modifiable risk factors associated with gastric cancer for White, Black, and Asian/Pacific Islander patients are shown in Tables 5, 6, 7, respectively.

**Table 5:** Modifiable risk factors for gastric cancer present in White patients

Modifiable risk factors for gastric cancer present in White patients	Group 1 (18-44)	Group 2 (45-64)	Group 3 (65 or more)	P Value
Helicobacter pylori	40 (2.35%)	170 (1.28%)	264 (1.08%)	0.107
History or current tobacco use	612 (35.88%)	7020 (53.13%)	11,525 (47.07%)	<0.01
Alcohol use	35 (2.06%)	637 (4.83%)	754 (3.07%)	<0.01
Gastric ulcer	115 (6.76%)	747 (5.66%)	1841 (7.53%)	<0.01
Gastric polyp	20 (1.18%)	79 (0.6%)	350 (1.43%)	<0.01
Obesity	166 (9.71%)	1691 (12.79%)	2265 (9.25%)	<0.01

**Table 6:** Modifiable risk factors for gastric cancer present in Black patients

Modifiable risk factors for gastric cancer present in Black patients	Group 1 (18-44)	Group 2 (45-64)	Group 3 (65 or more)	P Value
Helicobacter pylori	30 (3.57%)	130 (2.64%)	160 (2.43%)	0.68
History or current tobacco use	230 (27.38%)	2315 (46.96%)	2720 (41.27%)	<0.01
Alcohol use	20 (2.38%)	395 (8.01%)	185 (2.81%)	<0.01
Gastric ulcer	35 (4.17%)	525 (10.65%)	645 (9.79%)	0.02
Gastric polyp	5 (0.6%)	3 (0.06%)	105 (1.59%)	0.07
Obesity	55 (6.55%)	370 (7.51%)	490 (7.06%)	0.87

**Table 7:** Modifiable risk factors for gastric cancer present in Asian/Pacific Islander patients

Modifiable risk factors for gastric cancer present in Asian/Pacific Islander patients	Group 1 (18-44)	Group 2 (45-64)	Group 3 (65 or more)	P Value
Helicobacter pylori	50 (3.97%)	125 (2.72%)	105 (1.51%)	0.01
History or current tobacco use	370 (29.37%)	1626 (35.4%)	2350 (33.79%)	0.20
Alcohol use	20 (1.59%)	170 (3.7%)	145 (2.08%)	0.03
Gastric ulcer	105 (8.33%)	380 (8.28%)	780 (11.21%)	0.04
Gastric polyp	0 (0%)	45 (0.98%)	70 (1.01%)	0.28
Obesity	70 (5.56%)	260 (5.66%)	290 (4.17%)	0.26

## Discussion

Although overall rates of gastric cancer have been decreasing over the last several decades, rates of gastric cancer in young patients (under 45 years of age) in the United States are increasing. Several studies have suggested that overall rates of gastric cancer have decreased due to improvements in hygiene and refrigeration technology, resulting in a lower incidence of *H. pylori* infections and improved diets. The rise of gastric cancer in young populations has been hypothesized to result from increasing rates of obesity in Western populations [12]. There may also be a genetic component, as postulated by Ju et al., who demonstrated a distinct pattern of gene expression in Hispanic patients under 50 years of age compared to older patients. Younger patients showed upregulation of DNA replication, cell cycle, and DNA repair pathways, while older patients had upregulation of programmed cell death pathways [13].

Our study demonstrates that there is a higher rate of *H. pylori* infection and gastric cancer specifically among younger Hispanic patients when compared to other races and older age groups. Previous studies have also demonstrated disparities in gastric cancer, particularly in Hispanic patients. Narasimman et al. demonstrated that Hispanic patients had a higher incidence of early-onset gastric cancer compared to non-Hispanic individuals in both rural and urban areas [14]. A study by Toal et al. showed that Hispanic patients with gastric cancer have a higher prevalence of molecular subtypes associated with poorer prognosis compared to Asian and White patients [15]. Additionally, studies have demonstrated that outcomes also differ between Hispanic and non-Hispanic individuals. As detailed in a study by Zarrinkhoo et al., Hispanic patients with gastric cancer were more likely to be misdiagnosed despite no differences in insurance/primary care physician status, present with metastatic disease or peritoneal carcinomatosis, and had increased rates of mortality (65% vs. 35%, p <0.001) [16].

In this study, we found that high rates of gastric cancer in younger Hispanic individuals were correlated with high rates of *H. pylori *infection in a similar population, suggesting that there may be a role for tailored screening and treatment of asymptomatic individuals, particularly in this at-risk population. Currently, the American College of Gastroenterologists (ACG) has no recommendations on screening asymptomatic patients for *H. pylori *infection due to insufficient evidence [17]. Further research is needed to determine the risks and benefits of screening and offering appropriate treatment in this population with unique demographic, genetic, and molecular profiles [18]. Additionally, community outreach and patient education, with a focus on cultural competency and language accessibility, may be pivotal in enhancing awareness, early detection, and improved outcomes in these communities [19].

Limitations

Our study had several limitations, primarily due to the limitations of the data available via the NIS database. For example, there are no data regarding specific dietary habits among different populations, gene profiles, exercise habits, or other modifiable risk factors that could impact the development of gastric cancer, limiting the ability to further risk stratify the populations studied. Additionally, the NIS catalogs hospitalized patients only, limiting our cohort to patients who have been admitted and excluding those who were managed in an outpatient setting. Finally, in the NIS database, there is a new entry for each hospitalization, meaning that it is possible for one patient to be categorized in multiple entries if hospitalized more than once during the study period.

## Conclusions

In conclusion, our study highlights a significant trend of increasing rates of *H. pylori *infection and gastric cancer in young Hispanic individuals, which contrasts with global trends of decreasing rates of gastric cancer. This disparity emphasizes the need for further studies regarding targeted screening for this demographic. Further studies focusing on identifying other modifiable risk factors present in young Hispanics should be performed. For example, our study identified a high prevalence of tobacco use correlated with gastric cancer in this population.

A possible area of study that could have public health and policy implications could be investigating the impact of asymptomatic *H. pylori *screening in high-risk populations, such as Hispanics, and comparing this intervention to other ethnic groups to evaluate if there would be a benefit in the prevention of gastric cancer in the United States.

Despite the fact that current guidelines lack specific screening recommendations for *H. pylori *infection in asymptomatic individuals in the general population, our findings identify a possible demographic in which targeted screening, risk modification, and early intervention may improve outcomes.
